# VEGF‐A‐Dependent Vascular Signaling Modulates BMSC‐Derived Cartilaginous Organoid Fate During Endochondral Spinal Bone Regeneration

**DOI:** 10.1155/sci5/6327556

**Published:** 2026-11-15

**Authors:** Yinyu Wei, Jun Shang, Bizeng Zhao

**Affiliations:** ^1^ Department of Orthopedics Linfen People’s Hospital The Seventh Affiliated Hospital of Shanxi Medical University Linfen China lfsrmyy.cn; ^2^ Department of Orthopedics Shanghai Jiao Tong University Affiliated Sixth People’s Hospital Shanghai China sjtu.edu.cn

**Keywords:** cartilaginous organoids, endochondral ossification, spinal bone defect, vascular invasion, VEGF-A signaling

## Abstract

Spinal bone regeneration is hindered by early avascularity and incomplete integration with host bone. We investigated whether BMSC‐derived cartilaginous organoids could serve as developmental templates whose cartilage‐to‐bone transition is modulated by VEGF‐A‐associated vascular signaling. Mouse BMSCs were assembled into cartilaginous organoids and evaluated through in vitro vascular modulation, ectopic implantation, and a mouse spinal bone defect model. VEGF‐A signaling was manipulated using HUVEC coculture, recombinant VEGF‐A_165_, or VEGF‐A neutralization. The organoids progressively matured into hypertrophy‐competent cartilaginous tissues with enhanced matrix deposition and hypertrophic characteristics. HUVEC coculture and VEGF‐A stimulation enhanced vascular‐associated maturation and promoted the cartilage‐to‐bone transition, whereas VEGF‐A neutralization attenuated these effects. In vivo, VEGF‐A promoted vascularization, hypertrophic remodeling, ectopic mineralization, and bone formation, whereas anti‐VEGF preserved cartilage and reduced ossification. In spinal defects, organoid‐loaded GelMA enhanced bone regeneration and host–repair integration, with VEGF‐A further improving structural repair, whereas anti‐VEGF reduced regenerative outcomes. These findings suggest that VEGF‐A‐associated vascular signaling modulates cartilaginous organoid fate and supports developmental organoid grafting as a potential strategy for spinal bone regeneration. Further studies are warranted to clarify the relative contributions of endothelial‐associated and direct VEGF‐A responses, thereby refining the mechanistic understanding of vascular regulation during cartilaginous organoid maturation and endochondral bone regeneration.

## 1. Introduction

Spinal bone defects and inadequate spinal fusion remain major clinical challenges because successful repair requires not only the formation of mineralized tissue but also rapid vascularization, mechanical restoration, and continuous integration with the surrounding host bone [1]. Autologous bone grafting remains the clinical benchmark, yet its use is limited by donor‐site morbidity, restricted graft availability, and variable graft resorption and remodeling [2, 3]. Although allogeneic grafts and synthetic substitutes avoid donor‐site injury, they often provide insufficient biological activity and may result in delayed vascularization, fibrous tissue formation, or incomplete bone integration [4, 5]. Therefore, regenerative strategies capable of generating living, vascularized, and structurally integrated bone are urgently needed.

Conventional bone tissue engineering commonly relies on the direct osteogenic differentiation of bone marrow‐derived mesenchymal stem cells (BMSCs) before implantation [6]. However, osteogenically committed cells have relatively high metabolic demands and may exhibit poor survival within the hypoxic, metabolically stressed, and inflammatory environment of an early bone defect [7]. Moreover, the formation of mineralized tissue alone does not necessarily reproduce the organized remodeling and host integration observed during physiological bone healing [8]. These limitations indicate that recreating the developmental process of bone formation may be more effective than directly generating a terminal osteogenic construct in vitro.

Endochondral ossification provides a developmentally inspired pathway for bone regeneration [9, 10]. During skeletal development and fracture healing, a transient cartilage template forms before vascular invasion, hypertrophic maturation, matrix mineralization, and replacement by bone [11]. Cartilage‐like tissues are relatively tolerant of low oxygen tension and can therefore survive during the early avascular phase after implantation [12, 13]. Once invaded by blood vessels, the cartilage template can support matrix remodeling, osteogenic differentiation, and new bone formation [6]. Thus, engineered cartilage may serve as a more physiologically relevant intermediate for regenerating bone in poorly vascularized defects [14].

BMSC‐derived cartilaginous organoids offer a promising platform for implementing this strategy [15]. Compared with dispersed cells, organoids preserve cell–cell interactions, endogenous extracellular matrix, and spatially coordinated differentiation, allowing them to function as living developmental tissue units [16, 17]. However, a key scientific question remains unresolved: whether vascular invasion is merely associated with organoid ossification or acts as a causal regulator of the cartilage‐to‐bone transition [18, 19]. This distinction is particularly important because vascular invasion is generally considered undesirable in cartilage engineering, whereas the same process may be deliberately harnessed to drive endochondral bone regeneration [20].

Vascular endothelial growth factor (VEGF)‐mediated angiogenic signaling is positioned at the interface between vascular invasion, chondrocyte hypertrophy, matrix remodeling, and osteogenesis [21, 22]. Nevertheless, most previous studies have evaluated vascularization as an accompanying outcome rather than directly testing its regulatory role in organoid fate [23]. Therefore, it remains unclear whether experimentally manipulating VEGF‐A availability can directly influence the developmental trajectory of cartilaginous organoids from cartilage maturation toward endochondral bone formation. In the present study, we established an endothelial coculture system and modulated VEGF‐A signaling using recombinant VEGF‐A_165_ or the VEGF‐A‐neutralizing monoclonal antibody B20‐4.1.1 (anti‐VEGF). This bidirectional intervention enabled us to evaluate whether VEGF‐A‐associated vascular signaling, reflected by endothelial coculture responses and VEGF‐A/anti‐VEGF modulation, influences hypertrophic maturation, mineralization, and endochondral ossification of the organoids.

We therefore developed BMSC‐derived cartilaginous organoids as ossification‐competent developmental templates and evaluated their regenerative capacity through sequential in vitro, ectopic implantation, and spinal bone defect experiments. We hypothesized that VEGF‐A‐associated vascular signaling would regulate the transition of cartilaginous organoids toward hypertrophic maturation, mineralization, and endochondral bone formation. In this study, we investigated whether VEGF‐A‐associated vascular signaling influences the transition of BMSC‐derived cartilaginous organoids from cartilage maturation toward endochondral bone formation. Through in vitro vascular modulation, ectopic implantation, and spinal bone defect models, we evaluated how VEGF‐A availability affects organoid maturation, mineralization, and host–repair integration.

## 2. Materials and Methods

### 2.1. Experimental Design

This study was designed to determine whether vascular invasion regulates the transition of mouse BMSC‐derived cartilaginous organoids from a cartilage phenotype toward hypertrophic maturation, endochondral ossification, and spinal bone regeneration. The experimental workflow consisted of four sequential stages: generation and longitudinal characterization of cartilaginous organoids, in vitro modulation of VEGF‐A‐associated vascular signaling, evaluation of ectopic endochondral ossification after subcutaneous implantation in nude mice, and assessment of bone regeneration and host–repair integration in a mouse spinal bone defect model.

Five biological replicates were included for each in vitro condition or time point. For longitudinal diameter measurements, the same five individually indexed organoids were followed over time; all other time‐course assays used independently prepared samples at each time point.

### 2.2. Animals and Ethics Statement

Four‐week‐old male C57BL/6J mice were used as BMSC donors. Male C57BL/6J mice aged 8–10 weeks and weighing 22–26 g were used as recipients in the spinal bone defect model. Male BALB/c nude mice aged 6–8 weeks and weighing 18–22 g were used for ectopic implantation. All animals were obtained from Shanghai SLAC Laboratory Animal Co., Ltd. (Shanghai, China). Animals were housed under specific pathogen‐free conditions at 22 ± 2°C and 50%–60% relative humidity under a 12‐h light/dark cycle, with unrestricted access to standard chow and water.

All animal procedures were approved by the Institutional Animal Care and Use Committee of Shanghai Jiao Tong University Affiliated Sixth People’s Hospital and were conducted in accordance with institutional guidelines for the care and use of laboratory animals.

C57BL/6J mice were used as BMSC donors and as recipients in the spinal bone defect experiment to maintain a syngeneic cell‐transplantation background. BALB/c nude mice were used for the ectopic implantation experiment.

### 2.3. Isolation and Culture of Mouse BMSCs

Mouse BMSCs were isolated from the femurs and tibias of C57BL/6J mice. Six donor mice were euthanized under deep anesthesia, and the femurs and tibias were harvested under sterile conditions. After removal of the surrounding soft tissues, both epiphyses were excised, and the bone marrow cavities were flushed repeatedly with low‐glucose Dulbecco’s modified Eagle’s medium (DMEM; Gibco, Thermo Fisher Scientific, Waltham, MA, USA) using a 25‐gauge needle.

The resulting cell suspension was passed through a 70‐μm cell strainer and centrifuged at 300 × *g* for 5 min. The cell pellet was resuspended in low‐glucose DMEM supplemented with 10% fetal bovine serum (FBS), 1% penicillin–streptomycin, and 2 mM L‐glutamine (all from Gibco, USA). Cells were seeded into tissue culture flasks and maintained at 37°C in a humidified atmosphere containing 5% CO_2_.

Nonadherent cells were removed after 24 h, and the medium was subsequently replaced every 2–3 days. At approximately 80% confluence, adherent cells were detached using 0.25% trypsin‐EDTA (Gibco, USA) and passaged at a ratio of 1:3. Cells obtained from different donor mice were pooled at Passage 2 to reduce donor‐specific variability. BMSCs at Passages 3–5 were used for organoid generation.

### 2.4. Generation of Mouse BMSC‐Derived Cartilaginous Organoids

Mouse BMSCs were resuspended at 2.5 × 10^5^ cells/mL, and 100 *μ*L of cell suspension containing 2.5 × 10^4^ cells was added to each well of a round‐bottom 96‐well ultra‐low‐attachment plate (Corning, NY, USA). The plates were centrifuged at 300 × *g* for 5 min to facilitate cellular aggregation and incubated for 24 h in a complete growth medium.

The medium was subsequently replaced with serum‐free chondrogenic induction medium consisting of high‐glucose DMEM, 1% insulin–transferrin–selenium premix, 100 nM dexamethasone, 50 *μg*/mL L‐ascorbic acid 2‐phosphate, 40 *μ*g/mL L‐proline, 1 mM sodium pyruvate, 1% penicillin–streptomycin, and 10 ng/mL recombinant mouse transforming growth factor‐*β*3 (TGF‐*β*3; PeproTech/Thermo Fisher Scientific, USA).

One organoid was maintained in each microwell. Half of the culture medium was replaced every 2 days without disturbing the aggregates. Organoids were cultured for up to 28 days. Organoids showing fragmentation, marked asymmetry, or a diameter deviating by more than 20% from the batch mean were excluded before subsequent treatment.

### 2.5. Longitudinal Assessment of Organoid Morphology

The same five individually indexed organoids were followed longitudinally on Days 1, 3, 7, 14, and 28. Their positions within the 96‐well plate were recorded to ensure that the same organoids were imaged at each time point.

Bright‐field images were acquired using an inverted phase‐contrast microscope (Leica DMi8, Wetzlar, Germany) under identical magnification and illumination conditions. Two mutually perpendicular diameters passing through the center of each organoid were measured using ImageJ software, and their mean was recorded as the organoid diameter.

Because the same organoids were measured repeatedly at multiple time points, changes in organoid diameter over time were analyzed using a repeated‐measures statistical approach.

### 2.6. Histological and Biochemical Characterization of Organoids

Organoids collected at the indicated time points were fixed in 4% paraformaldehyde for 24 h, embedded in 2% agarose, dehydrated through a graded ethanol series, embedded in paraffin, and sectioned at a thickness of 5 *μ*m. Sections were stained with hematoxylin and eosin (H&E), Safranin O/Fast Green, and Toluidine Blue using commercial staining kits according to the manufacturer’s instructions.

For biochemical analysis, organoids collected at the indicated time points were digested overnight in papain solution at 60°C. Sulfated glycosaminoglycan (GAG) content was measured using the dimethylmethylene blue method, and collagen Type II (COL II) content was quantified using a mouse COL II enzyme‐linked immunosorbent assay kit (Beyotime, China). Total protein content was determined using a bicinchoninic acid assay, and GAG and COL II contents were normalized to total protein content.

### 2.7. Human Umbilical Vein Endothelial Cell (HUVEC) Culture

HUVECs (ScienCell Research Laboratories, Carlsbad, CA, USA) were maintained in endothelial cell medium (ScienCell) supplemented with 5% FBS, 1% endothelial cell growth supplement, and 1% penicillin–streptomycin. Cells were cultured at 37°C in a humidified atmosphere containing 5% CO_2_, and the culture medium was replaced every 2 days. HUVECs at Passages 3–6 were used for coculture experiments. Only cultures displaying typical cobblestone‐like morphology and a viability greater than 90%, as determined by trypan blue exclusion, were used.

### 2.8. *In Vitro* Vascular Modulation

Day‐14 cartilaginous organoids were transferred to hypertrophic/mineralization medium consisting of high‐glucose DMEM, 1% insulin–transferrin–selenium, 50 *μ*g/mL L‐ascorbic acid 2‐phosphate, 10 mM *β*‐glycerophosphate, 1 nM triiodothyronine, 100 nM dexamethasone, and 1% penicillin–streptomycin.

The organoids were assigned to four experimental conditions: (1) control, organoids cultured alone; (2) +HUVEC, organoids cocultured with HUVECs; (3) +VEGF, organoids cocultured with HUVECs and treated with recombinant VEGF‐A_165_; and (4) +anti‐VEGF, organoids cocultured with HUVECs and treated with B20‐4.1.1, a VEGF‐A‐neutralizing monoclonal antibody.

HUVECs were added at 1 × 10^4^ cells per organoid in the +HUVEC, +VEGF, and +anti‐VEGF groups. Five organoids were cultured in each well of an ultra‐low‐attachment 24‐well plate containing 1 mL of coculture medium. The coculture medium consisted of hypertrophic/mineralization medium and endothelial cell medium mixed at a ratio of 1:1. The same basal medium composition was used for all four groups.

Recombinant VEGF‐A_165_ was added at a final concentration of 50 ng/mL. B20‐4.1.1, a VEGF‐A‐neutralizing monoclonal antibody, was added at a final concentration of 10 *μ*g/mL and was operationally designated anti‐VEGF throughout the manuscript. The medium was replaced every 2 days, and freshly prepared VEGF‐A_165_ or B20‐4.1.1 was added at each medium change. The treatment period was 14 days.

### 2.9. Alkaline Phosphatase (ALP) and Alizarin Red S Assays

ALP staining was performed using a BCIP/NBT ALP staining kit according to the manufacturer’s instructions. For quantitative analysis, organoids were lysed, and ALP activity was measured using a p‐nitrophenyl phosphate‐based assay. Absorbance was measured at 405 nm, and ALP activity was normalized to total protein content determined using a bicinchoninic acid assay.

Matrix mineralization was evaluated by Alizarin Red S (ARS) staining. Organoids were fixed in 4% paraformaldehyde and stained with 2% ARS solution adjusted to pH 4.2. After removal of unbound dye, the stained organoids were washed with distilled water. For quantitative analysis, the bound dye was extracted using 10% cetylpyridinium chloride, and absorbance was measured at 450 nm.

After 14 days of vascular modulation, mineralized organoid samples were fixed in 4% paraformaldehyde and scanned using a SkyScan 1276 micro–computed tomography (CT) system. The scanning voltage, current, filter, voxel size, reconstruction parameters, mineralization threshold, and region‐of‐interest definition were kept constant among groups. The total segmented mineralized tissue volume was quantified using CTAn software.

### 2.10. Immunofluorescence Staining and Image Analysis

Paraffin sections were deparaffinized, rehydrated, and subjected to heat‐mediated antigen retrieval in citrate buffer at pH 6.0. Sections were permeabilized with 0.2% Triton X‐100 and blocked with 5% bovine serum albumin for 1 h at room temperature. The sections were subsequently incubated overnight at 4°C with primary antibodies against collagen Type I (COL I), COL II, aggrecan (ACAN), collagen type X (COL X), runt‐related transcription factor 2 (RUNX2), osteocalcin (OCN), or CD31. After washing, the sections were incubated with the appropriate Alexa Fluor‐conjugated secondary antibodies, and nuclei were counterstained with DAPI.

Mouse CD31 was detected on paraffin sections using a rabbit monoclonal anti‐CD31 antibody (Cell Signaling Technology; clone D8V9E; Catalog No. 77699), followed by fluorescence‐conjugated secondary antibody detection. Images for the same marker were acquired using identical microscope, magnification, exposure, and acquisition settings. For organoid sections, the entire organoid cross‐sectional area was defined as the region of interest. For ectopic and spinal tissue sections, five nonoverlapping fields within the predefined repair or interface region were analyzed. Measurements from the five fields were averaged to generate one value per biological replicate and were not treated as independent observations.

### 2.11. Preparation of Organoid‐Loaded Gelatin Methacryloyl (GelMA) Constructs

GelMA (Engineering for Life, Suzhou, China) was dissolved in sterile PBS at 60°C to obtain a 10% weight/volume precursor solution. Lithium phenyl‐2,4,6‐trimethylbenzoylphosphinate was added at 0.25% weight/volume as the photoinitiator. The precursor solution was sterilized using a 0.22‐μm filter and maintained at 37°C before use.

GelMA hydrogel was selected as a biocompatible and photocrosslinkable matrix for organoid encapsulation and delivery. In this study, GelMA served primarily as a three‐dimensional supportive scaffold to maintain construct integrity and localization after implantation, rather than as an independent osteoinductive intervention.

For ectopic implantation, each construct contained 30 Day‐21 cartilaginous organoids suspended in 50 *μ*L of GelMA. For the mouse spinal bone defect model, each construct contained 12 Day‐21 cartilaginous organoids suspended in approximately 2 *μ*L of GelMA. For the VEGF groups, recombinant VEGF‐A_165_ was incorporated at a total dose of 250 ng per ectopic construct and 100 ng per spinal construct. For the anti‐VEGF groups, B20‐4.1.1, a mouse IgG2a monoclonal antibody that neutralizes human and mouse VEGF‐A, kindly provided by Genentech (South San Francisco, CA, USA), was incorporated at a total dose of 20 *μ*g per ectopic construct and 8 *μ*g per spinal construct. Blank constructs contained the corresponding volume of GelMA without organoids or vascular modulators. The organoid suspension was gently mixed to achieve an even distribution. Ectopic constructs were transferred into sterile molds, whereas spinal constructs were prepared as approximately 2‐*μ*L microconstructs. The GelMA constructs were photocrosslinked using 405‐nm light at approximately 10 mW/cm^2^ for 3 min. Constructs were implanted within 2 h after preparation.

### 2.12. Subcutaneous Ectopic Implantation in Nude Mice

Twenty male BALB/c nude mice were sequentially allocated to four groups, with five mice per group:

1. organoid,
2. organoid + VEGF,
3. organoid + anti‐VEGF, and
4. blank GelMA.

Anesthesia was induced using 3% isoflurane (RWD Life Science, Shenzhen, China) and maintained with 1.5%–2.0% isoflurane in oxygen. After shaving and disinfecting the dorsal skin, an approximately 8‐mm longitudinal incision was made. A subcutaneous pocket was created by blunt dissection lateral to the incision, and one construct was inserted into the dorsal pocket of each mouse.

The incision was closed using 5–0 absorbable sutures. Meloxicam was administered subcutaneously at 2 mg/kg immediately after surgery and once daily for 2 consecutive days. Animals were monitored daily for wound healing, infection, implant extrusion, and abnormal behavior.

Four weeks after implantation, mice were deeply anesthetized and euthanized according to the approved institutional protocol. Explants were harvested with a narrow margin of surrounding tissue. Each mouse contributed to one independent biological replicate.

### 2.13. Mouse Spinal Bone Defect Model

Thirty male C57BL/6J mice, aged 8–10 weeks and weighing 22–26 g, were sequentially allocated to six groups, with five mice per group:

1. defect (left untreated),
2. GelMA,
3. GelMA + organoid,
4. GelMA + organoid + VEGF,
5. GelMA + organoid + anti‐VEGF, and
6. autograft.

Anesthesia was induced with 3% isoflurane and maintained with 1.5%–2.0% isoflurane. Mice were placed in the prone position, and the lumbar region was shaved and disinfected. A posterior midline incision was made over the L3‐L5 vertebral levels. The paraspinal muscles were separated carefully to expose the L4 posterior element while preserving the facet capsules and dura.

A standardized full‐thickness rectangular defect measuring 1.5 mm in length and 1.0 mm in width was created in the L4 lamina using a low‐speed dental drill under continuous sterile saline irrigation. The underlying dura was carefully preserved. Care was taken to avoid penetration of the dura or thermal injury to the surrounding bone.

The defect was left untreated in the defect group. In the GelMA group, approximately 2 *μ*L of blank GelMA construct was placed into the defect. In the organoid‐based treatment groups, approximately 2 *μ*L of GelMA construct containing 12 Day‐21 cartilaginous organoids alone, with VEGF‐A_165_, or with B20‐4.1.1, as appropriate, was placed into the defect. In the autograft group, autologous bone particles collected during preparation of the L4 laminar defect were packed into the defect until it was filled.

The muscles and skin were closed separately using 5–0 absorbable sutures. Cefazolin was administered intraperitoneally at 20 mg/kg immediately before surgery and once daily for 2 days. Buprenorphine was administered subcutaneously at 0.05 mg/kg every 12 h for 48 h after surgery. Mice were allowed unrestricted cage activity after recovery.

Animals were euthanized at 8 weeks, and the operated lumbar spinal segments were collected en bloc. Thereafter, individual specimens were allocated to one of five mutually exclusive downstream processing routes: decalcified paraffin histology and immunofluorescence, undecalcified fluorochrome analysis, or biomechanical testing. The number of animals analyzed for each endpoint is specified in the corresponding figure legend.

### 2.14. Sequential Fluorochrome Labeling

Dynamic mineralization was evaluated by sequential fluorochrome labeling. Calcein (Sigma‐Aldrich, St. Louis, MO, USA) was administered intraperitoneally at 10 mg/kg 14 days before euthanasia. Alizarin Red complexone (Sigma‐Aldrich, St. Louis, MO, USA) was administered intraperitoneally at 30 mg/kg 3 days before euthanasia.

After collection, specimens designated for fluorochrome analysis were fixed in 70% ethanol without decalcification, dehydrated in graded ethanol, and embedded in methyl methacrylate. Sections were cut and ground to a final thickness of approximately 20–30 *μ*m using an EXAKT cutting and grinding system.

Calcein fluorescence was visualized using the green channel, whereas Alizarin Red fluorescence was visualized using the red channel. All images were acquired using identical exposure parameters.

### 2.15. Micro‐CT Analysis

Ectopic explants and spinal specimens designated for histological analysis were fixed in 4% paraformaldehyde before micro‐CT scanning. Specimens designated for undecalcified fluorochrome analysis were fixed in 70% ethanol. Specimens designated for biomechanical testing were scanned without chemical fixation while maintained in a hydrated state and were subsequently stored at −20°C until testing.

Ectopic explants were scanned at 50 kV and 200 *μ*A using a 0.5‐mm aluminum filter, a rotation step of 0.4°, and an isotropic voxel size of 9 *μ*m.

Mouse spinal specimens were scanned at 70 kV and 200 *μ*A using a 0.5‐mm aluminum filter, a rotation step of 0.4°, and an isotropic voxel size of 9 *μ*m. Hydroxyapatite phantoms with known mineral densities were scanned under identical conditions for bone mineral density (BMD) calibration.

Images were reconstructed using NRecon software with uniform beam‐hardening and ring‐artifact corrections. Morphometric analysis was performed using CTAn software, and three‐dimensional images were generated using CTVol software.

For ectopic explants, the entire implant was defined as the region of interest, and bone volume fraction (BV/TV) was calculated as mineralized bone volume divided by total construct volume.

For spinal specimens, a manually contoured three‐dimensional region of interest corresponding to the original rectangular defect was defined using consistent anatomical landmarks. The same anatomical boundaries and mineralization threshold were applied to all specimens. BV/TV, BMD, trabecular number (Tb.N), and trabecular thickness (Tb.Th) were calculated.

### 2.16. Histological Processing

Following micro‐CT scanning, specimens intended for paraffin histology were decalcified in 10% EDTA at pH 7.4. The decalcification solution was replaced every 2–3 days. Ectopic explants were decalcified for approximately 2 weeks, whereas mouse spinal specimens were decalcified for approximately 3–4 weeks.

Samples were dehydrated, cleared, embedded in paraffin, and sectioned at 5 *μ*m. Ectopic explants were stained with H&E and Safranin O/Fast Green. Spinal specimens were stained with H&E, Toluidine Blue, and Safranin O/Fast Green.

Three nonconsecutive central sections separated by at least 100 *μ*m were evaluated for each specimen. Measurements obtained from the sections of the same animal were averaged to produce one biological value.

### 2.17. Histomorphometric Analysis of the Host–Repair Interface

The host–repair interface was defined as the boundary between native host bone and the implanted or regenerated repair tissue.

Bone–repair contact was calculated as

(1) bone−repair contact%=length of direct bone contacttotal evaluated interface length×100.

Fibrous tissue area was calculated as

(2) fibrous tissue area %=fibrous tissue area within the interface ROItotal interface ROI area×100.

Three central sections were analyzed for each animal, and the section‐level values were averaged.

### 2.18. Pull‐Out Testing

After micro‐CT scanning, spinal specimens designated for biomechanical analysis were wrapped in PBS‐moistened gauze and stored at −20°C. Before testing, samples were thawed at room temperature and kept hydrated throughout preparation.

The vertebral body was embedded in polymethylmethacrylate and fixed in a custom fixture connected to a universal mechanical testing system (Instron 5943, Instron, Norwood, MA, USA). The repaired posterior element was secured to the loading arm using a custom micro‐clamp. The loading direction was aligned perpendicular to the host–repair interface.

A preload of 0.2 N was applied to remove slack. Displacement‐controlled tensile loading was subsequently performed at 0.5 mm/min until failure of the repaired interface. Force–displacement data were continuously recorded. The maximum force before interfacial failure was defined as maximum pull‐out force.

### 2.19. Statistical Analysis

Statistical analyses were performed using GraphPad Prism 10. Data are presented as mean ± standard deviation (SD), and *p* < 0.05 was considered statistically significant.

Because the same five individually indexed organoids were followed longitudinally for the diameter measurements shown in Figure 1C, these data were analyzed using one‐way repeated‐measures analysis of variance (ANOVA), with the Geisser–Greenhouse correction applied when the sphericity assumption was violated, followed by Tukey’s multiple‐comparisons test. GAG content, COL II content, and immunofluorescence outcomes obtained from independently prepared organoid samples at different time points were analyzed using ordinary one‐way ANOVA followed by Tukey’s multiple‐comparisons test.

**FIGURE 1:**
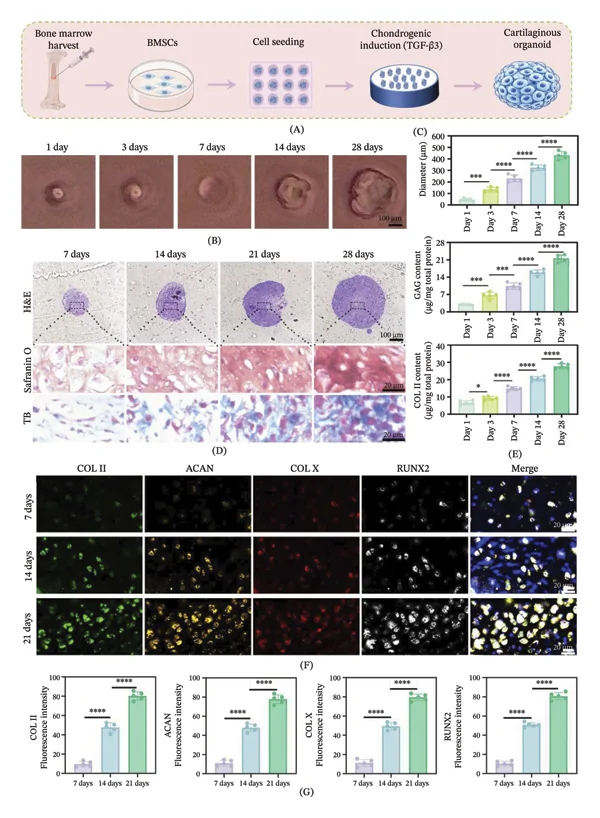
Construction and characterization of BMSC‐derived cartilaginous organoids. (A) Schematic illustration of the generation of BMSC‐derived cartilaginous organoids. Bone marrow was harvested for the isolation and expansion of BMSCs. BMSCs were subsequently seeded into multiwell plates to form cellular aggregates and cultured in a chondrogenic induction medium containing transforming growth factor‐*β*3 (TGF‐*β*3) to generate cartilaginous organoids. (B) Representative bright‐field images showing the morphological evolution of cartilaginous organoids on Days 1, 3, 7, 14, and 28. (C) Quantification of organoid diameter at the indicated culture time points. (D) Representative hematoxylin and eosin (H&E), Safranin O, and Toluidine Blue (TB) staining images of cartilaginous organoids on Days 7, 14, 21, and 28. (E) Quantification of glycosaminoglycan (GAG) and collagen Type II (COL II) contents in the organoids on Days 1, 3, 7, 14, and 28. (F) Representative immunofluorescence images of COL II, aggrecan (ACAN), collagen type X (COL X), and runt‐related transcription factor 2 (RUNX2) in cartilaginous organoids on Days 7, 14, and 21. Nuclei were counterstained with DAPI. (G) Quantification of COL II, ACAN, COL X, and RUNX2 fluorescence intensities at the indicated time points. Data are presented as mean ± SD; error bars represent SD. For (C), the same five individually indexed organoids were followed longitudinally (*n* = 5) and analyzed using one‐way repeated‐measures ANOVA followed by Tukey’s multiple‐comparisons test. For (E and G), *n* = 5 independently prepared biological samples per time point; ordinary one‐way ANOVA followed by Tukey’s multiple‐comparisons test was used. ^∗^ *p* < 0.05, ^∗∗∗^ *p* < 0.001, and ^∗∗∗∗^ *p* < 0.0001.

For the in vitro vascular modulation experiment, CD31‐positive area, ALP activity, ARS absorbance, and mineralized tissue volume were analyzed using ordinary one‐way ANOVA followed by Tukey’s multiple‐comparisons test. COL II, COL X, RUNX2, and OCN expressions between the +VEGF and +anti‐VEGF groups were compared using an unpaired two‐tailed Student’s *t*‐test for each outcome.

Quantitative outcomes from the ectopic implantation and spinal bone defect experiments were analyzed using ordinary one‐way ANOVA followed by Tukey’s multiple‐comparisons test. Statistical significance is indicated as follows: ^∗^ *p* < 0.05, ^∗∗^ *p* < 0.01, ^∗∗∗^ *p* < 0.001, and ^∗∗∗∗^ *p* < 0.0001; ns indicates no statistically significant difference.

## 3. Results

### 3.1. BMSC‐Derived Organoids Acquired a Cartilage‐Rich and Hypertrophy‐Competent Phenotype

BMSCs were assembled into cellular aggregates and cultured in a chondrogenic induction medium containing TGF‐*β*3 to generate cartilaginous organoids (Figure 1A). Bright‐field imaging showed progressive enlargement and morphological consolidation of the organoids from Day 1 to Day 28 (Figure 1B). Consistently, organoid diameter increased significantly over time, with the largest dimensions observed on Day 28 (Figure 1C).

Histological analysis revealed progressive extracellular matrix deposition and tissue organization during culture. H&E staining showed progressive organoid enlargement and increasingly compact tissue organization, whereas Safranin O and Toluidine Blue staining demonstrated gradual accumulation of a proteoglycan‐rich cartilage matrix between Days 7 and 28 (Figure 1D). Biochemical measurements further showed time‐dependent increases in GAG and COL II contents (Figure 1E).

Immunofluorescence analysis demonstrated that COL II and ACAN expression increased markedly from Day 7 to Day 21, confirming progressive acquisition of a cartilaginous phenotype (Figure 1F,G). COL X and RUNX2 also increased during this period, indicating the acquisition of a hypertrophic and osteogenic‐associated phenotype while the organoids retained abundant cartilage matrix. Together, these findings showed that chondrogenically induced BMSC aggregates developed into cartilage‐rich organoids with the capacity to undergo subsequent endochondral maturation.

### 3.2. VEGF‐A‐Associated Vascular Signaling Altered Hypertrophic Maturation and Mineralization *In Vitro*

To determine whether VEGF‐A‐associated vascular signaling influences organoid maturation, organoids were cultured alone or cocultured with HUVECs. HUVEC‐containing cocultures were further treated with VEGF or anti‐VEGF. HUVEC coculture increased the CD31‐positive area relative to organoids cultured alone. VEGF‐A further enhanced this response, whereas anti‐VEGF markedly reduced CD31 positivity (Figure 2A,B).

**FIGURE 2:**
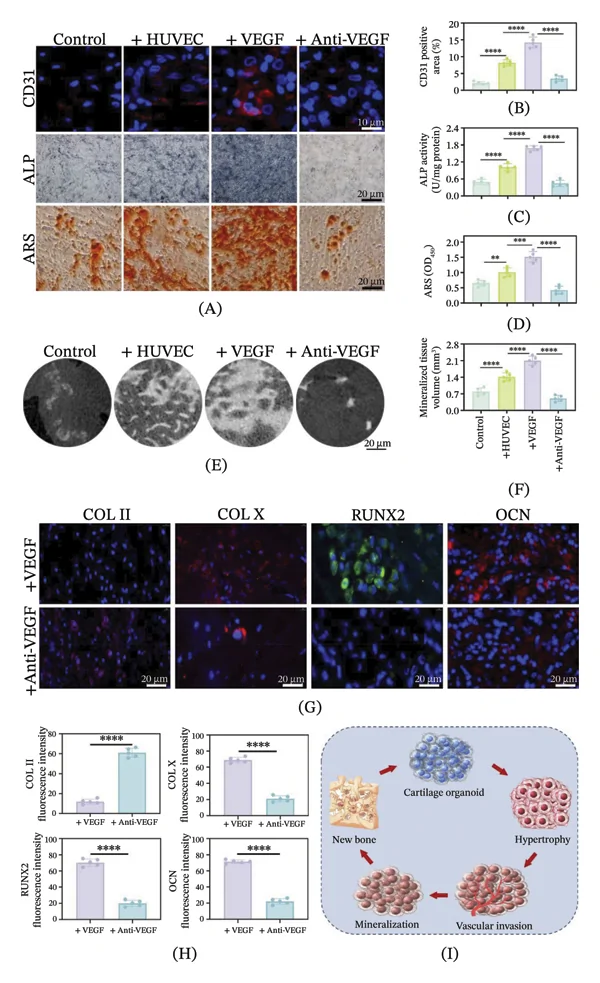
Vascular stimulation promotes hypertrophic maturation and endochondral ossification of BMSC‐derived cartilaginous organoids in vitro. (A) Representative images of CD31 immunofluorescence, alkaline phosphatase (ALP) staining, and Alizarin Red S (ARS) staining of cartilaginous organoids cultured alone (control), cocultured with human umbilical vein endothelial cells (HUVECs; +HUVEC), cocultured with HUVECs and treated with recombinant VEGF‐A_165_ (+VEGF), or cocultured with HUVECs and treated with B20‐4.1.1, a VEGF‐A‐neutralizing monoclonal antibody (+anti‐VEGF). Nuclei in the immunofluorescence images were counterstained with DAPI. (B) Quantification of the CD31‐positive area in the four experimental groups. (C) Quantitative analysis of ALP activity. (D) Quantification of ARS staining by measuring absorbance at 450 nm. (E) Representative microcomputed tomography (micro‐CT) images of mineralized organoids under the indicated culture conditions. (F) Quantitative micro‐CT analysis of mineralized bone‐like tissue volume. (G) Representative immunofluorescence images of collagen Type II (COL II), collagen type X (COL X), runt‐related transcription factor 2 (RUNX2), and osteocalcin (OCN) in VEGF‐A‐treated and anti‐VEGF‐treated organoids. Nuclei were counterstained with DAPI. (H) Quantification of COL II, COL X, RUNX2, and OCN fluorescence intensities in the +VEGF and +anti‐VEGF groups. (I) Schematic illustration of the proposed sequence of vascular invasion‐coupled endochondral ossification, including cartilage organoid formation, hypertrophic maturation, vascular invasion, matrix mineralization, and new bone formation. Data are presented as mean ± SD; error bars represent SD; *n* = 5 independent samples per group. Ordinary one‐way ANOVA followed by Tukey’s multiple‐comparisons test was used for (B–D) and (F), and an unpaired two‐tailed Student’s *t*‐test was used for (H). ^∗∗^ *p* < 0.01, ^∗∗∗^ *p* < 0.001, and ^∗∗∗∗^ *p* < 0.0001.

A similar treatment‐dependent pattern was observed for osteogenic maturation. Compared with HUVEC coculture alone, VEGF‐A treatment further increased ALP activity and ARS‐detectable mineral deposition, whereas anti‐VEGF treatment reduced both outcomes (Figure 2A,C,D). Micro‐CT analysis similarly showed that VEGF‐A increased mineralized tissue volume relative to HUVEC coculture alone, whereas anti‐VEGF reduced mineralized tissue formation (Figure 2E,F).

VEGF‐A and anti‐VEGF exerted opposing effects on organoid phenotype. VEGF‐A‐treated organoids exhibited lower COL II expression but markedly higher COL X, RUNX2, and OCN expression than anti‐VEGF‐treated organoids (Figure 2G,H). Conversely, VEGF‐A neutralization preserved COL II expression while suppressing hypertrophic and osteogenic markers. These results showed that VEGF‐A‐treated organoids exhibited reduced cartilage‐associated marker expression and increased hypertrophic and osteogenic‐associated marker expression compared with anti‐VEGF‐treated organoids (Figure 2I).

### 3.3. VEGF‐A Availability Affected Ectopic Endochondral Ossification *In Vivo*

To test whether the in vitro findings translated to an in vivo environment, cartilaginous organoids were encapsulated in GelMA and implanted subcutaneously into nude mice. Organoid, organoid + VEGF, organoid + anti‐VEGF, and blank GelMA constructs were harvested 4 weeks after implantation (Figure 3A).

**FIGURE 3:**
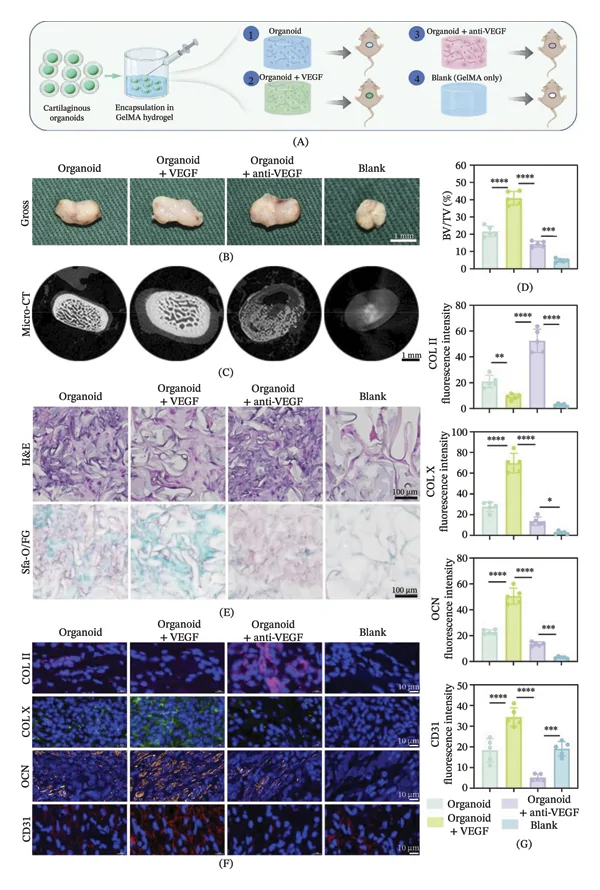
Vascularization regulates ectopic endochondral ossification of BMSC‐derived cartilaginous organoids in vivo. (A) Schematic illustration of the ectopic implantation procedure. Cartilaginous organoids were encapsulated in GelMA hydrogel and assigned to four treatment groups: organoid, organoid + VEGF, organoid + anti‐VEGF, and blank. The constructs were harvested 4 weeks after implantation for subsequent analyses. (B) Representative gross photographs of explants from the cartilaginous organoid, organoid + VEGF, organoid + anti‐VEGF, and blank groups after ectopic implantation. (C) Representative micro‐CT images of the explants from the indicated groups. (D) Quantitative micro‐CT analysis of bone volume fraction (BV/TV) within the explants. (E) Representative H&E and Safranin O/Fast Green (Saf O/FG) staining images showing tissue morphology, residual cartilage matrix, and newly formed bone within the explants. (F) Representative immunofluorescence images of collagen Type II (COL II), collagen type X (COL X), osteocalcin (OCN), and CD31 in the explants. Nuclei were counterstained with DAPI. (G) Quantification of COL II, COL X, OCN, and CD31 fluorescence intensities in the four experimental groups. Data are presented as mean ± SD; error bars represent SD; *n* = 5 animals per group. Ordinary one‐way ANOVA with Tukey’s multiple‐comparisons test was used. ^∗^ *p* < 0.05, ^∗∗^ *p* < 0.01, ^∗∗∗^ *p* < 0.001, and ^∗∗∗∗^ *p* < 0.0001.

Gross examination showed distinct differences in explant development among the four groups (Figure 3B). Micro‐CT revealed extensive mineralized tissue in the organoid + VEGF group, whereas the organoid group exhibited an intermediate degree of mineralization. Anti‐VEGF treatment markedly reduced mineralized tissue formation, and little mineralization was detected in blank GelMA constructs (Figure 3C). Quantitative analysis confirmed that BV/TV was highest in the organoid + VEGF group, lower in the organoid group, and substantially reduced by anti‐VEGF treatment (Figure 3D).

Histological staining demonstrated abundant remodeled and newly mineralized tissue in VEGF‐A‐treated explants. The organoid group contained both cartilage‐like matrix and newly formed tissue, consistent with ongoing endochondral remodeling. In comparison, explants in the organoid + anti‐VEGF group retained more cartilage‐like matrix and showed less extensive bone formation, whereas blank GelMA exhibited minimal osteochondral tissue development (Figure 3E).

Immunofluorescence analysis further demonstrated opposing cartilage and bone phenotypes. Anti‐VEGF treatment maintained the strongest COL II signal, while VEGF‐A treatment reduced COL II and markedly increased COL X, OCN, and CD31 expression (Figure 3F,G). The organoid group exhibited intermediate levels of hypertrophic, osteogenic, and vascular markers. These findings showed that VEGF‐A‐dependent vascularization promoted ectopic cartilage remodeling and bone formation, whereas VEGF‐A neutralization maintained a more cartilaginous phenotype and attenuated ossification.

### 3.4. Cartilaginous Organoids Promoted Structural and Functional Repair of Spinal Bone Defects

The regenerative capacity of the organoid constructs was subsequently evaluated in a spinal bone defect model (Figure 4A). At 8 weeks after implantation, three‐dimensional micro‐CT reconstructions showed limited mineralized tissue and incomplete defect bridging in the defect and GelMA groups. These findings indicated that GelMA alone provided a supportive matrix but was insufficient to independently induce substantial osteogenic regeneration. The GelMA + organoid group showed increased new bone formation and greater structural continuity within the defect. The GelMA + organoid + VEGF group exhibited further enhancement of mineralized bone formation and defect bridging, whereas these regenerative effects were attenuated in the GelMA + organoid + anti‐VEGF group. The autograft group also exhibited extensive defect repair (Figure 4B).

**FIGURE 4:**
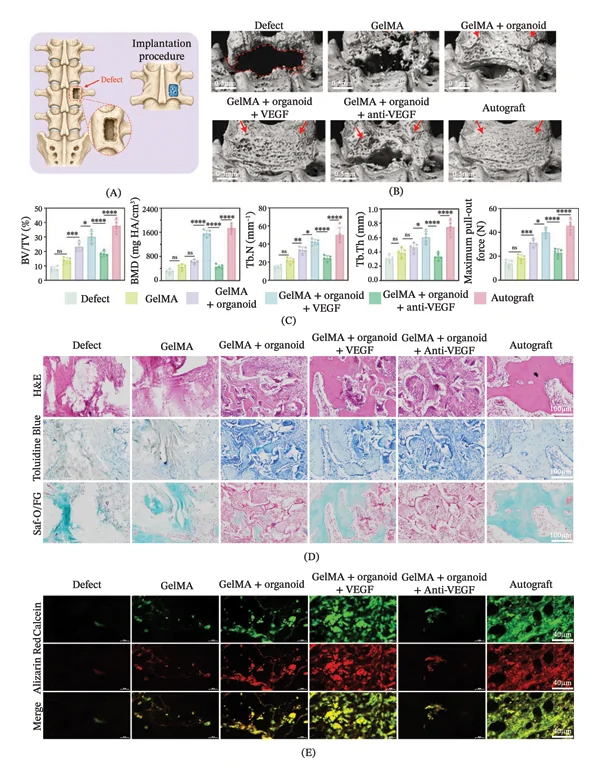
BMSC‐derived cartilaginous organoids promote spinal bone defect repair through VEGF‐A‐dependent endochondral ossification. (A) Schematic illustration of the mouse spinal bone defect model and implantation procedure. A standardized defect was created in the L4 posterior spinal element and treated according to the following six groups: defect, GelMA, GelMA + organoid, GelMA + organoid + VEGF, GelMA + organoid + anti‐VEGF, and autograft. (B) Representative three‐dimensional micro‐CT reconstructions of the operated spinal segments at 8 weeks after surgery. The red arrows indicate newly formed mineralized tissue and bone bridging within the defect region. (C) Quantitative analyses of bone volume fraction (BV/TV), bone mineral density (BMD), trabecular number (Tb.N), trabecular thickness (Tb.Th), and maximum pull‐out force in the six experimental groups. (D) Representative H&E, Toluidine Blue, and Saf‐O/FG staining images of the spinal defect region at 8 weeks after surgery, showing newly formed bone, residual cartilage‐like matrix, fibrous tissue, and structural continuity within the repaired region. (E) Representative fluorescence images of sequential calcein and Alizarin Red complexone labeling. Calcein fluorescence is shown in green, Alizarin Red fluorescence is shown in red, and overlapping mineralization fronts are shown in the merged images. Quantitative data in (C) are presented as mean ± SD; error bars represent SD; *n* = 5 animals per group. BV/TV, BMD, Tb.N, Tb.Th, and maximum pull‐out force were analyzed using ordinary one‐way ANOVA followed by Tukey’s multiple‐comparisons test. “ns” indicates no statistically significant difference; ^∗^ *p* < 0.05, ^∗∗^ *p* < 0.01, ^∗∗∗^ *p* < 0.001, and ^∗∗∗∗^ *p* < 0.0001.

Quantitative micro‐CT analyses supported these observations. GelMA + organoid increased BV/TV, BMD, Tb.N, and Tb.Th compared with the defect and GelMA groups. VEGF‐A supplementation further improved these microarchitectural outcomes and reduced the difference relative to the autograft group, although the autograft group retained higher values for the measured parameters. By contrast, the GelMA + organoid + anti‐VEGF group showed lower bone volume, mineral density, and trabecular microarchitectural parameters than the GelMA + organoid + VEGF and GelMA + organoid groups (Figure 4C).

Biomechanical testing showed that the structural improvements were accompanied by increased maximum pull‐out force. Organoid‐containing constructs produced greater mechanical fixation than untreated defects or GelMA alone, while VEGF‐A generated the strongest performance among the organoid‐based groups. Anti‐VEGF treatment attenuated the biomechanical benefit of organoid implantation (Figure 4C).

Histological examination confirmed limited tissue continuity and greater fibrous tissue formation in the defect and GelMA groups. Organoid‐loaded GelMA promoted cartilage‐associated matrix remodeling and new bone formation, and VEGF‐A supplementation produced the most extensive mineralized tissue and structural continuity among the engineered constructs. Anti‐VEGF treatment resulted in less mature repair tissue and reduced bone formation (Figure 4D). Sequential calcein and Alizarin Red labeling showed active mineral deposition in the organoid group, with the greatest fluorochrome incorporation observed after VEGF‐A treatment. Mineralization was substantially weaker after anti‐VEGF treatment (Figure 4E). Collectively, these results demonstrated that cartilaginous organoids promoted both structural and functional spinal bone repair through a VEGF‐A‐sensitive process.

### 3.5. Organoid Treatment Enhanced Vascularized Integration at the Host–Repair Interface

Histological analysis of the host–repair interface revealed limited direct bone contact and prominent intervening fibrous tissue in the defect and GelMA groups (Figure 5A). GelMA + organoid treatment increased continuity between the host bone and repair tissue. VEGF‐A further enhanced interface organization and newly formed bone, whereas anti‐VEGF reduced tissue continuity and increased the persistence of nonmineralized interface tissue. The autograft group showed extensive bone integration.

**FIGURE 5:**
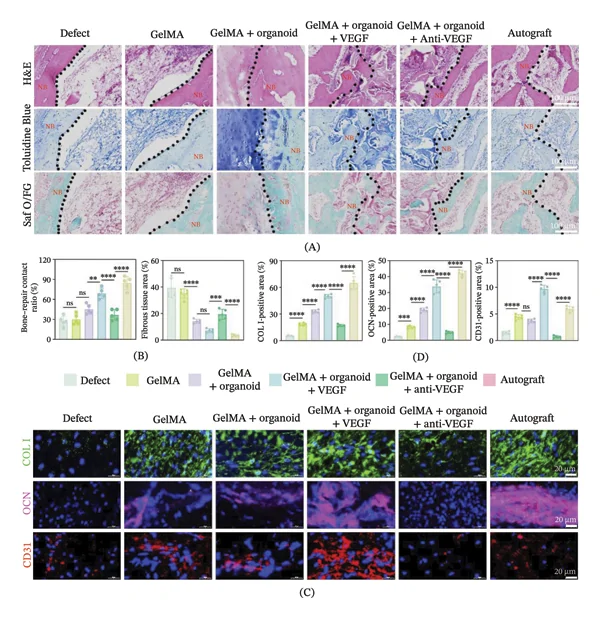
Cartilaginous organoids enhance new bone formation and integration at the host–repair interface. (A) Representative H&E, Toluidine Blue, and Saf‐O/FG staining images of the host–repair interface at 8 weeks after surgery in the defect, GelMA, GelMA + organoid, GelMA + organoid + VEGF, GelMA + organoid + anti‐VEGF, and autograft groups. Black dotted lines delineate the host–repair interface, and “NB” indicates newly formed bone. (B) Histomorphometric quantification of bone–repair contact ratio and fibrous tissue area at the host–repair interface. (C) Representative immunofluorescence images of collagen Type I (COL I), OCN, and CD31 in the six experimental groups. Nuclei were counterstained with DAPI. (D) Quantification of COL I‐positive area, OCN‐positive area, and CD31‐positive area at the host–repair interface. Data are presented as mean ± SD; error bars represent SD; *n* = 5 animals per group. Bone–repair contact ratio, fibrous tissue area, COL I‐positive area, OCN‐positive area, and CD31‐positive area were analyzed separately using ordinary one‐way ANOVA followed by Tukey’s multiple‐comparisons test. “ns” indicates no statistically significant difference; ^∗∗^ *p* < 0.01, ^∗∗∗^ *p* < 0.001, and ^∗∗∗∗^ *p* < 0.0001.

Histomorphometric analysis demonstrated that organoid implantation increased bone–repair contact ratio and reduced fibrous tissue area compared with the defect and GelMA groups. VEGF‐A treatment produced the highest bone–repair contact ratio and the lowest fibrous tissue area among the engineered treatment groups, whereas the autograft group showed the highest overall bone–repair contact ratio (Figure 5B).

Immunofluorescence staining showed weak COL I, OCN, and CD31 expression in untreated defects. Organoid implantation increased COL I and OCN expression relative to the defect and GelMA groups, whereas the increase in CD31 was most pronounced after VEGF‐A supplementation. VEGF‐A supplementation generated the strongest COL I, OCN, and CD31 signals among the engineered treatments, while anti‐VEGF substantially reduced their expression (Figure 5C,D). These findings indicated that VEGF‐A‐associated vascular signaling was associated with increased bone formation within the defect and the establishment of a vascularized and mineralized connection between the engineered tissue and host bone.

### 3.6. Proposed Mechanism of VEGF‐A‐Associated Vascular Signaling During Organoid Ossification

The combined findings are consistent with a staged model in which BMSC‐derived cartilaginous organoids initially function as cartilage templates after implantation. VEGF‐A‐associated vascular signaling is associated with enhanced hypertrophic differentiation and cartilage matrix remodeling, followed by mineralization, new bone formation, and interface integration. The resulting tissue subsequently undergoes remodeling, characterized by increased bone contact, decreased fibrous tissue formation, and improved host–graft continuity (Figure 6). Thus, the results suggest that VEGF‐A‐associated vascular signaling contributes to the transition from cartilage maintenance to endochondral bone formation.

**FIGURE 6:**
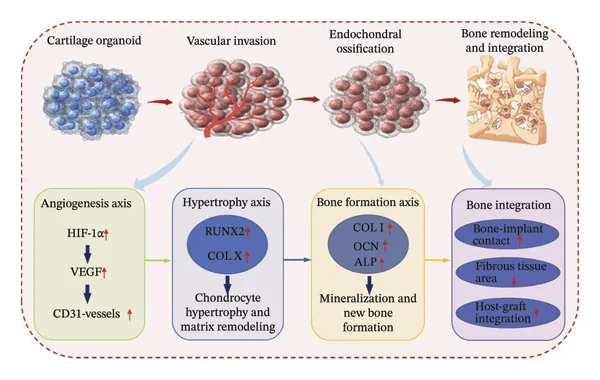
Proposed model illustrating the experimentally supported and hypothesized mechanisms underlying VEGF‐A‐dependent vascular signaling during endochondral ossification and bone integration mediated by BMSC‐derived cartilaginous organoids. Following implantation, BMSC‐derived cartilaginous organoids serve as developmental cartilage templates for endochondral bone formation. VEGF‐A‐dependent signaling promotes the invasion of CD31‐positive vessels into the organoids. Vascular invasion is associated with increased COL X and RUNX2 expression, indicating hypertrophic maturation and cartilage matrix remodeling. Subsequent increases in ALP activity, OCN expression, and COL I deposition promote matrix mineralization and new bone formation. The newly formed bone subsequently undergoes remodeling and integration with the surrounding host tissue, resulting in increased bone–repair contact, reduced fibrous tissue formation, and enhanced host–repair integration. Upward and downward arrows indicate relative increases and decreases, respectively.

## 4. Discussion

The present study developed BMSC‐derived cartilaginous organoids as developmental templates for spinal bone regeneration and identified VEGF‐A‐associated vascular signaling as an important contributor to their cartilage‐to‐bone transition. Rather than representing a terminal cartilage or osteogenic construct, these organoids occupy an intermediate developmental state that preserves cartilage characteristics while retaining the capacity for subsequent hypertrophic maturation and endochondral ossification. This developmental flexibility may provide advantages over direct osteogenic strategies, particularly in environments where early vascular supply is limited. These effects were reproduced in an ectopic implantation model and subsequently translated into improved bone formation, microarchitecture, mechanical performance, and host–repair integration in a spinal defect model. Together, the data support a developmental grafting strategy in which vascularization is used not merely to sustain implanted cells but to program organoid fate.

A central feature of this approach is the use of a cartilage intermediate rather than a terminally osteogenic construct. Conventional cell‐based bone engineering frequently attempts to generate osteoblast‐like cells or mineralized matrices before implantation [24, 25]. Such constructs may be vulnerable to the initially hypoxic and mass‐transport‐limited environment of a bone defect and may not fully reproduce the sequential remodeling events of physiological bone formation [26]. In contrast, cartilaginous tissues naturally function in relatively avascular environments and provide an extracellular matrix capable of undergoing hypertrophic remodeling and mineral replacement [27, 28]. The simultaneous increase in COL II, ACAN, COL X, and RUNX2 during organoid maturation indicated that the constructs developed a cartilage‐rich but ossification‐competent state [29]. This dual phenotype is important because an insufficiently chondrogenic construct may lack a stable developmental template, whereas an excessively stable cartilage phenotype may resist conversion into bone.

The opposing effects of VEGF‐A supplementation and VEGF‐A neutralization indicate that VEGF‐A availability influences the balance between cartilage maintenance and hypertrophic progression. Rather than simply increasing tissue formation, bidirectional VEGF‐A manipulation altered the developmental trajectory of the organoids toward either cartilage preservation or endochondral maturation. These findings suggest that vascular stimulation does not simply increase the overall quantity of tissue; rather, it alters the developmental state of the organoids from cartilage maintenance toward hypertrophy and ossification. The similar, although generally weaker, effects produced by HUVEC coculture further support the contribution of endothelial–organoid interactions.

The use of B20‐4.1.1 as anti‐VEGF strengthens the gain‐ and loss‐of‐function framework because the antibody directly neutralizes extracellular VEGF‐A [30]. Nevertheless, the present data do not completely distinguish endothelial‐mediated effects from direct VEGF‐A actions on organoid cells. BMSCs and differentiating chondrocytes may respond to VEGF‐A‐dependent signals independently of newly formed vessels, while endothelial cells may provide additional paracrine factors beyond VEGF‐A [31, 32]. Accordingly, the findings support a VEGF‐A‐dependent vascularization mechanism but do not establish that every observed change is exclusively mediated by endothelial invasion. Accordingly, the present design could not completely separate endothelial‐mediated effects from direct VEGF‐A responses in organoid cells. Future studies incorporating endothelial‐free VEGF‐A stimulation, endothelial‐conditioned medium experiments, or cell‐specific VEGF‐A manipulation will be required to further dissect the relative contributions of endothelial‐associated and direct VEGF‐A signaling pathways. Although GelMA served as a supportive carrier that enabled organoid implantation and localization, the present study was not designed to investigate GelMA as an independent biomaterial platform. The regenerative effects observed in this study were primarily attributed to the biological activity of VEGF‐A‐modulated organoids, as supported by comparisons with blank GelMA controls. It should also be noted that the HIF‐1α‐related pathway depicted in the proposed model was not directly examined in the present study. Therefore, this component should be interpreted as a mechanistic hypothesis based on previous reports linking hypoxia‐responsive signaling, angiogenesis, and endochondral ossification rather than as a demonstrated pathway in our experimental system.

The ectopic implantation experiment provided an intermediate validation step between in vitro maturation and functional spinal repair by demonstrating that VEGF‐A modulation influenced the ossification trajectory of implanted organoids in vivo. This reciprocal pattern is consistent with a vascularization‐coupled endochondral transition in which cartilage preservation and bone formation represent dynamically regulated states rather than independent outcomes [33, 34]. The minimal mineralization in blank GelMA constructs further indicated that the hydrogel alone was insufficient to reproduce the osteogenic response generated by the organoids.

The spinal defect model demonstrated that the developmental regulation of organoid maturation could be translated into functionally relevant repair in a mechanically demanding environment. Compared with ectopic implantation, spinal reconstruction requires not only mineralized tissue formation but also structural continuity and mechanical integration with the host skeleton. The improved repair response following VEGF‐A modulation indicates that controlling the maturation trajectory of cartilaginous organoids may enhance their ability to undergo endochondral bone formation and restore functional defects. Importantly, the consistency among imaging, histological, fluorochrome, and biomechanical assessments supports that the regenerated tissue achieved meaningful structural and functional integration rather than mineral deposition alone.

Successful spinal bone regeneration requires continuous osseous bridging and stable integration between the repair tissue and the host skeleton [35, 36]. The organoid‐containing constructs enhanced host–repair continuity and reduced fibrous separation at the interface, suggesting that developmental maturation of the graft contributes not only to intragraft ossification but also to functional integration with surrounding bone. These effects were most pronounced after VEGF‐A treatment and were attenuated by anti‐VEGF. The interface findings therefore extend the proposed mechanism beyond intragraft ossification. Vascular stimulation appears to facilitate not only bone formation within the construct but also coordinated remodeling and integration at the boundary with host bone. This distinction is clinically important because a mineralized implant that remains separated from the host by fibrous tissue may fail despite appearing radiopaque on imaging.

The methodological innovation of this study lies in the integration of organoid engineering with bidirectional vascular manipulation across sequential experimental levels. Unlike previous osteochondral studies that exploited native vascular gradients to generate spatially heterogeneous tissues, the present study directly modulated VEGF‐A availability to regulate cartilaginous organoid fate in a spinal bone defect model and linked this transition to interfacial mechanical fixation and host–repair integration. Rather than treating vascularization as a secondary endpoint, we enhanced VEGF‐A‐dependent endothelial signaling through HUVEC coculture and exogenous VEGF‐A stimulation and suppressed it using anti‐VEGF treatment. The same biological relationship was then examined during in vitro maturation, ectopic ossification, spinal defect repair, and host–repair integration. This hierarchical design provides stronger mechanistic support than a single terminal comparison and positions the organoids as tunable developmental grafts whose fate can be directed by the vascular microenvironment.

Several limitations should be acknowledged. First, the in vivo analyses were confined to terminal endpoints; therefore, the temporal order of vascular invasion, cartilage hypertrophy, matrix resorption, and bone deposition could not be resolved directly. Second, donor and host cell contributions to the regenerated tissue were not distinguished. Third, because an isotype‐matched antibody group was not included, nonspecific effects associated with antibody incorporation cannot be completely excluded. Fourth, the local release profiles of VEGF‐A and anti‐VEGF from GelMA were not characterized, and the effective exposure of the implanted tissues therefore remains uncertain. Fifth, the inflammatory microenvironment after spinal defect surgery was not directly characterized in this study. Macrophage polarization and inflammatory cytokine profiles may influence angiogenesis and osteogenesis during bone regeneration, and future studies incorporating immune profiling will be required to determine whether VEGF‐A‐dependent vascular regulation is also associated with modulation of inflammatory responses. Finally, the in vivo findings were obtained from murine models and should therefore be interpreted within a preclinical context.

## 5. Conclusion

In conclusion, mouse BMSC‐derived cartilaginous organoids formed cartilage‐rich, hypertrophy‐competent developmental templates capable of undergoing endochondral bone formation after implantation. VEGF‐A‐associated vascular signaling was associated with enhanced endothelial responses, hypertrophic maturation, matrix mineralization, new bone formation, and host–repair integration, whereas anti‐VEGF treatment preserved the cartilaginous phenotype and attenuated ossification. In the mouse spinal bone defect model, organoid‐loaded GelMA improved bone microarchitecture, dynamic mineralization, interfacial mechanical fixation, and bone–repair contact while reducing fibrous tissue formation. Collectively, these findings support a regulatory role for VEGF‐A‐associated vascular signaling in modulating cartilaginous organoid maturation and highlight developmental organoid grafting as a potential strategy for vascularized and integrated spinal bone regeneration.

## Author Contributions

Yinyu Wei: investigation, formal analysis, and writing–original draft. Jun Shang: conceptualization, supervision, funding acquisition, and writing–review and editing. Bizeng Zhao: conceptualization, supervision, and writing–review and editing.

## Funding

This work was funded by the Fundamental Research Program of Shanxi Province (Grant No. 202403021221346), the Scientific Research Project on Traditional Chinese Medicine of Linfen City (Grant No. LF2025ZYYA002), the Science and Technology Medical Innovation Project under the Foundation Strengthening Project of Shanxi Provincial Health Commission (Grant No. 2026QJGC023).

## Disclosure

All authors read and approved the final manuscript.

## Conflicts of Interest

The authors declare no conflicts of interest.

## Data Availability

The data supporting the findings of this study are available from the corresponding author upon reasonable request.
